# Prevalence of Lumpy Skin Disease in Africa: A Systematic Review and Meta-Analysis from 2007 to 2023

**DOI:** 10.1155/2024/9991106

**Published:** 2024-06-05

**Authors:** Balemual Abebaw

**Affiliations:** Department of Veterinary Science, College of Agriculture and Environmental Science, Debre Tabor University, Debre Tabor, Ethiopia

## Abstract

**Background:**

Lumpy skin disease (LSD) is an economically important, transboundary viral disease of cattle caused by LSD virus. This systematic review and meta-analysis are concerned with summarizing the status of lumpy skin disease in Africa.

**Methods:**

Literature searches between 2007 and 2023 were conducted using different database searches: Google, Google Scholar, PubMed, and Science Direct, and then the required information were extracted. A random effect model was used to pool estimates of prevalence at 95% CI. Publication bias was assessed by applying funnel plots.

**Results:**

The pooled estimated prevalence of LSD was 54% (95% CI: −0.09–1.18), with individual study prevalence ranging from 6 to 89%. Subgroup analysis showed that the random pooled prevalence of LSD was 28% (95% CI: −1.48–2.03) in Ethiopia, 37% (95% CI: −3.79–4.52) in Uganda, 88% (95% CI: −0.11–1.28) in Zimbabwe, and 58% (95% CI: −6.96–8.72) in Egypt. No single study was documented from western and northern Africa.

**Conclusion:**

The estimated pooled LSD prevalence was investigated as high and indicated nonsignificant variation among study countries. Thus, vaccination of cattle, quarantine, and biting insect control should be applied to control the disease.

## 1. Introduction

Lumpy skin disease (LSD) is an economically important, transboundary viral disease of cattle caused by LSD virus or prototype strain of Neethling virus in the genus *Capripoxvirus*, family *Poxviridae* [1, 2]. Lumpy skin disease viruses (LSDVs) have deoxyribose nucleic acid (DNA) of about 151 kb in size, consists of a central coding region connected by 2.4 kb similar inverted terminal repeats, and contains 156 putative genes [3, 4]. The hosts of LSDV are cattle and buffalo. The transmission occurs mechanically via blood-feeding insects or flies such as *Stomoxys calcitrans* and *Biomyia fasciata* [5, 6]. *Rhipicephalus appendiculatus ticks* can also transmit mechanically LSDV [7]. Warm humid agro-climate, watering, and introduction of new animals in a herd are risk factors for the spread of LSDV [8]. Rainy seasons and cow grazing along water areas lead to outbreaks of LSD. The herd size and low immunity were linked to the development of disease [9]. The temporal pattern of LSD disease occurrence can be explained with short-term, cyclical seasonal, and long-term trends [10]. LSD was announced for the first time in Zambia in 1929 [11–13]. It was described at the time due to its panzootic occurrence in Botswana, Zimbabwe, and the Republic of South Africa in 1943 and 1945 [14, 15]. The distribution of LSDV has reached from sub-Saharan countries to Egypt, and Western Africa [14]. It occurred in Kenya and was associated with an outbreak of sheep pox in 1957 [4, 8, 16]. LSDV was first indicated in Nigeria in 1974 and 1976 [17]. LSD was first explained in Egypt at the Suez and Ismailia provinces in May 1989 due to imported cattle from Africa [2, 5, 18]. LSD was reported for the first time in 1983 in the western part of Ethiopia. It has now spread to all regions and agro-climatic zones in the country [14, 19]. The first appearance of LSD outside Africa was reported in Kuwait in 1986 [16]. LSDV infection had been reported in Saudi Arabia in 1992 [20]. LSDV has spread since 2000 to the Middle East countries including Iran, Syria, and Israel [19]. In European countries, many outbreaks have been reported recently: in Croatia in 2016, the Balkan Area, Serbia in 2016, and Greece in 2015 [21].

LSD can be found in acute, subacute, or chronic forms with varying severity depending on the host breed and immunity of the host [13, 22]. LSD is identified by fever, nodular eruption all over the body, keratitis, conjunctivitis, and enlarged superficial lymph nodes [19, 23]. Diagnosis of LSDV is carried out based on clinical signs, virus neutralization test (VNT), indirect fluorescent antibody test (IFAT), virus isolation, and molecular techniques [1, 9]. IFAT has good sensitivity, but some rarely occurring Parapox and Orthopox virus antibodies may cross-react and influence its specificity during low serum dilution [14, 24]. The VNT is used mostly for detecting Capripox-specific antibodies. It has high specificity, which is useful to exclude false positives, but its sensitivity may be lower [9, 14]. Therefore, the most suggestive method for identifying such viruses is the polymerase chain reaction (PCR) technique [25]. Most of the molecular methods target Gprotein-coupled chemokine receptor (GPCR), P32, and RPO30 genes to distinguish and characterize LSDV and other Capri poxviruses. The GPCR gene is a suitable target for genetic identification between Capripoxviruses [19, 26]. The economic significance of LSD concerns production losses [14, 27]. LSD affects the African country's growth by causing decreases in milk yield, loss of draught power from lameness, abortion, and infertility in cows [22, 25]. Milk yield can be reduced by > 50% in infected herds and secondary infections can lead to mastitis. LSD can cause skin lesions and result in permanent scars on the hide, decreasing its value [7, 28]. LSD has low mortality <10% and varying morbidity [19, 29]. The morbidity and mortality of LSD vary considerably, depending on the type of breed, immunological status of the host, and the insect vectors [19, 25].

The control and prevention strategies for LSD include livestock movement controls, vaccination, vector control, quarantines, and the slaughter of infected and exposed animals [17, 25]. Vaccination is an appropriate strategy for preventing LSD [6, 20, 25]. Several live-attenuated capripoxvirus (CaPV) vaccine strains including the Neethling strain of LSDV, Kenyan sheep pox viruses (SPPV) and goat pox viruses (GTPV), Yugoslavian strain of SPPV, Romanian strain of SPPV, and Gorgan strain of GTPV are practiced for the prevention and control of LSD [1, 20]. LSD has been reported in different African countries with various prevalence rates due to the availability of warm humid agro-climate, watering, and the presence of vector and introduction of new animals in a herd as risk factors for the spread of LSDV [19, 28]. However, there has been no systematic review and pooled quantitative presentation of the status of LSD in Africa, which are essential in making strategies for preventing and controlling LSD to decrease its prevalence. Therefore, the objective of this systematic review and meta-analysis will be to determine the pooled prevalence of LSD in cattle in Africa.

## 2. Methods

### 2.1. Study Protocols

This systematic review and meta-analysis study was done by observing the PRISMA guideline [21]. The PRISMA checklist indicates the inclusion criteria for relevant information to this study. The objective of this study is to estimate the pooled prevalence of LSD in Africa. The study protocol had been registered on PROSPERO with reference number ID: CRD513988 and is available from: https://www.crd.york.ac.uk/ID=CRD513988.

### 2.2. Selection of Studies

The database search was carried out by using Science Direct, Scopus, Google Scholar, and PubMed/PubMed Central until June 29, 2023. The article search was done by using scientific terms. The keywords were “Lumpy Skin Disease” or “LSD,” “Sero-prevalence” or “Prevalence,” “Cattle” or “Bovine,” and “Africa.” We used “or” and “Boolean operators to identify studies with any of the keywords in their titles, abstracts, and full texts.”

### 2.3. Eligibility Criteria

The inclusion criteria to assure the eligibility of the searched articles includes: (1) research articles published in a reputable journal, which were conducted in Africa; (2) retrospective, prospective, and cross-sectional studies that presented the prevalence of LSD with endemic or outbreak occurrence; (3) articles with full information; (4) studies were carried out by using ELISA, IFAT, PCR, VNT, and SNT; (5) studies explained the total sample size and number of positive samples; (6) articles published in the English language, and (7) Articles published between 2007 and June 29, 2023. Articles that did not meet the above criteria were excluded.

### 2.4. Selection of Study and Data Extraction

Articles collected from different databases were exported to Mendeley software version 1.19. Repeated documents were identified and removed with Mendeley. The authors screened the titles and abstracts based on the predefined inclusion criteria. Similarly, the data extraction format was carried out concerning author, publication year, study year, study country, study design, disease occurrences, total sample, diagnostic test, and positive samples, individual-level prevalence.

The effect size and confidence intervals were calculated from the extracted data. Mendeley version 1.19.8 was used to manage citations. Microsoft Excel was used to code and manage all extracted data.

### 2.5. Quality Assessment

Independent researchers assessed the quality of the included articles using an assessment checklist. The checklist contained items assessing objectives and the methods parts (study design, sample size, disease occurrence, and statistical methods).

### 2.6. Statistical Analysis

The random-effects model meta-analysis was used to estimate the pooled prevalence of LSD at 95% CI [10, 30]. Heterogeneity (*I*^2^) among studies were evaluated. The *I*^2^ values of 25, 50, and 75% were classified as low, moderate, and high heterogeneity [15]. The *I*^2^ values (0%) tell us the absence of heterogeneity [15]. Subgroup analysis was calculated associated with country, study design, disease occurrence, and type of diagnostic method. Publication bias was evaluated using Begg and Egger's test [11] and by observing the funnel plot. Heterogeneity among studies also was evaluated using meta-regression. Country, study design, disease occurrence, event, and sample were evaluated by using multivariable meta-regression analysis. All statistical analyses were performed by using Stata/MP 17.0.

## 3. Results

### 3.1. Descriptive Results

**Figure 1:**
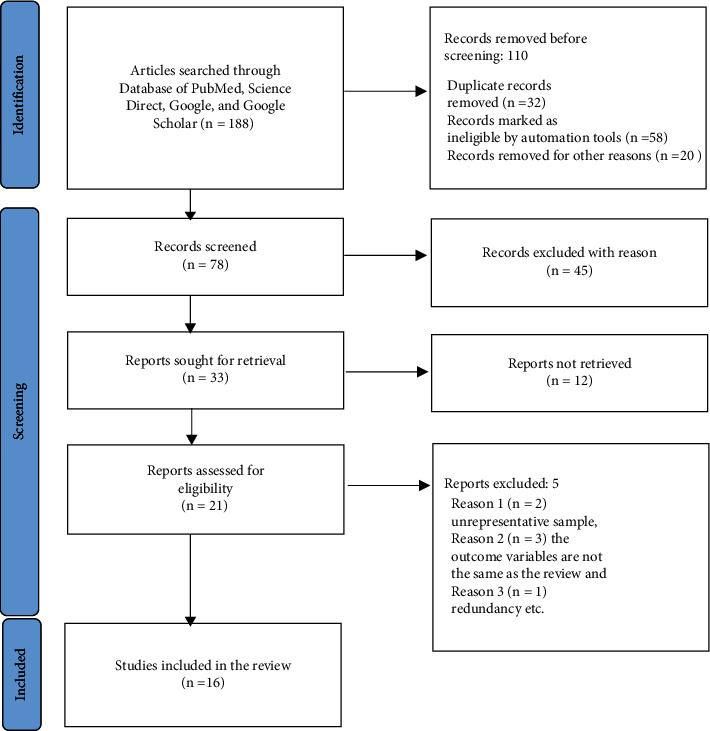
PRISMA 2020 flow diagram of the selection of eligible studies for quantitative analysis.

Among the 188 studies that were assembled, 16 articles met the inclusion criteria (Figure 1). The total sample size was 10090, and 1874 were identified as positive cases for the LSD virus. The pooled prevalence of LSD was 54% (95% CI: −0.09–1.18). The largest sample size is 2368 while the smallest sample size is 20 (Table 1). The LSD was identified in Egypt, Ethiopia, Uganda, and Zimbabwe.

### 3.2. Meta-Analysis

**Figure 2:**
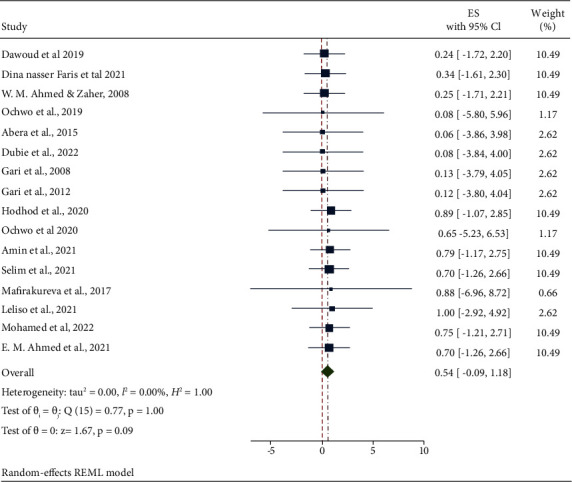
Forest plot illustrating the estimated pooled prevalence of LSD in Africa in different studies from 2007 to 2023.

The pooled estimated prevalence of LSD in Africa was 54% (95% CI: −0.09–1.18). The individual studies prevalence were ranged from 6.4 to 100%. Studies were approximately weighted nearly equal with an individual weight ranging from 1.17 to 10.49%. The forest plot describes the pooled prevalence of LSD in Africa as shown in Figure 2.

### 3.3. Subgroup Meta-Analysis

**Figure 3:**
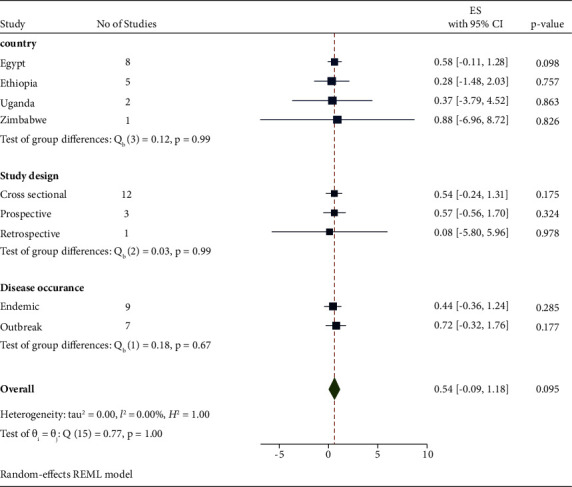
Subgroup meta-statics by study country, study design, and disease occurrence.

A subgroup meta-analysis was calculated for the country, disease occurrence, study design, and diagnostic methods (Figure 3). The sub-total estimated pooled prevalence of LSD by disease occurrence was 43.6% (95% CI: −0.364–1.236) in the endemic area and 71. 9% (95% CI: −0.324–1.761) in the outbreak condition (Table 2). The subgroup meta-analysis was also calculated for different countries. The highest prevalence was 88% with 95% CI (−6.96–8.72) in Zimbabwe, while the lowest prevalence was 27.7% CI (−1.476–2.03) in Ethiopia. The subgroup analysis has shown that many studies were reported from Egypt and Ethiopia.

The sub-total estimated pooled prevalence of LSD by study design was 53.6% (95% CI: −0.238–1.309) in cross-sectional, 57% (95% CI: −0.562–1.701) in perspective, and 8.1% (95% CI: −0.5.799–5.961) in retrospective. It indicated that many study designs were cross-sectional. The subgroup meta-analysis indicated that different types of diagnostic methods were applied for the examination of the LSD virus of which PCR was mostly applied having pooled prevalence at 72.6% (95% CI: −0.604–2.055).

### 3.4. Meta-Regression

Country, study design, disease occurrence, event, and sample were evaluated by using meta-regression analysis. None of the above variables was significantly related to the prevalence of LSD during multivariable meta-regression analysis (Table 3).

### 3.5. Publication Bias

**Figure 4:**
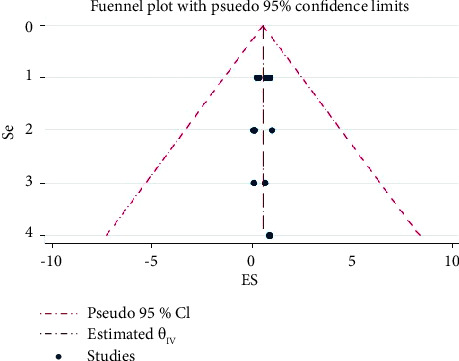
A funnel plot was used to illustrate the presence of publication bias in different studies.

There is a publication bias for the prevalence of LSD based on the analysis of effect sizes against standard errors. So, the funnel plot indicated that there are asymmetry patterns in studies assessing the presence of publication bias (Figure 4).

## 4. Discussion

This type of systematic review and meta-analysis has not been done on the prevalence of LSD in Africa until now. The results illustrated in this meta-analysis were from studies of LSD prevalence at different country levels between the years 2007 and 2023.

The meta-analysis of the pooled prevalence of LSD was estimated by using sixteen articles, which were published in different African countries. Among them, eight records were from Egypt, five from Ethiopia, two from Uganda, and one from Zimbabwe. Relatively higher studies were from Egypt. On the other hand, lower studies were from Uganda and Zimbabwe. Among the number of studies conducted, the cross-sectional study design type was used at the highest rate with a pooled prevalence of fifty-four percent (Table 2). Retrospective study designs were used at a lower rate with an effect size of eight percent. Most studies indicated that the LSD disease occurrence was due to endemic with a pooled prevalence of forty-four percent. Different types of diagnostic techniques were applied for LSD detection in this meta-analysis from which PCR was used at the highest rate and SNT, IFAT, and RT-PCR were used at the lowest rate.

LSD may be introduced to different countries by the movement of infected animals, biting insects that have fed on infected cattle, and importation of contaminated hides.

The random-effects model meta-analysis illustrated that there is no significant difference among studies with heterogeneity (*I*^2^), which tells us the existence of the LSD virus in different countries due to the movement of biting insects. Even though there was considerable homogeneity among studies, the weight of studies was not nearly equal. No variables, that were presented in the meta-regression, were statistically significant in describing the study's homogeneity.

**Figure 5:**
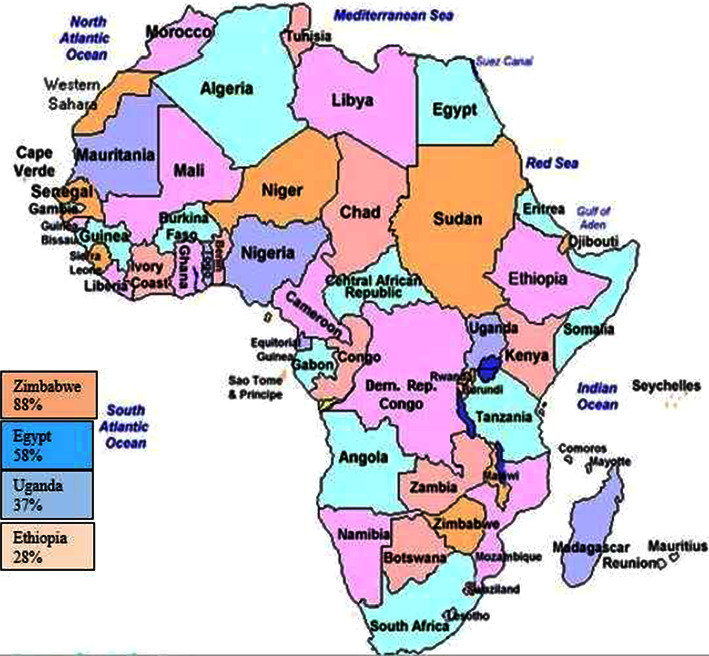
Pooled countries with the highest and lowest prevalence of LSD in Africa.

There is a higher prevalence of LSD in and Egypt while the lowest prevalence of LSD is in Ethiopia and Uganda (Figure 5). The pooled prevalence of LSD at all country level is 54%. The prevalence of LSD differs significantly among countries, which might be presented by the number of articles. This significant variation in the prevalence of LSD among African countries are based on the following reason: the availability of warm humid agro-climate, rainy season immunological status of the host, and the presence of vector and introduction of new animals in a herd as risk factors for the spread of LSDV [28, 30, 31]. Type of diagnostic method used, study design, disease occurrence and the number of studies reported are also the other the main determinant. Based on the current findings, LSD studies have been mostly done in Egypt [16, 20, 26]. In addition to this, a higher number of articles were documented in Ethiopia [14, 18]. A higher number of reports were presented from Egypt; the lowest prevalence was documented in Ethiopia. The highest prevalence of LSD was indicated in Zimbabwe. On the opposite side, no study was carried out from the western and northern parts of Africa.

There is a publication bias for the prevalence of LSD based on the analysis of effect sizes against standard errors (Figure 4). This review is an indicator of more task requirements for LSD studies in Africa. LSD affects the country's growth by causing decreases in milk yield, loss of draught power from lameness, abortion, and infertility in cows [10, 25, 29]. Milk yield can be reduced by > 50% in infected herds and secondary infections [14, 30, 31].

### 4.1. Limitations of the Study

A few numbers of published articles were selected from the countries involved in this study, and published articles from the North Africa and West Africa countries were not included about the prevalence of LSD in Africa.

## 5. Conclusions

This type of systematic review and meta-analysis has not been done on the prevalence of LSD in Africa until now. It indicated the existence of a high-pooled prevalence of LSD in Africa, and this disease had indicated nonsignificant variation among study countries. A higher number of reports were registered in Egypt and Ethiopia. The number of studies is limited about the prevalence of LSDV in Africa. Therefore, a further investigation had better to be carried out for LSD. Special strategies and policies are needed from each country's responsible bodies concerning Introduction of new cattle, the vaccination of cattle, and biting insects controlling to reduce the risk of LSD transmissions.

## Figures and Tables

**Table 1:** Summary of metadata of LSD from 2007 to 2023 published studies in Africa.

Author publishing year	Country	Region/district	Year of study	Study design	Disease occurrence	Sample size	Positive sample	APP	Type of diagnostic test
Dina Nasser Faris et al., 2021	Egypt	El-Qalyubia governorate	2018	Cross-sectional	Endemic	665	230	0.345	I ELISA
Ahmed and Zaher, 2008	Egypt	Lower Egypt	2007	Prospective	Endemic	640	163	0.2547	I ELISA
Ochwo et al., 2019	Uganda	Central, east, north, and west of Uganda	2016	Retrospective	Outbreak	2263	185	0.087	I ELISA
Abera et al., 2015	Ethiopia	Western Wollega of Oromiya	2012	Cross-sectional	Endemic	544	35	0.0643	IFAT
Dubie et al., 2022	Ethiopia	Afar region	2018	Cross-sectional	Endemic	384	29	0.076	SNT
Gari et al., 2008	Ethiopia	Wello zone of Amahara and Borena zone of Oromiya	2007	Cross-sectional	Endemic	1712	216	0.126	IFAT and VNT
Gari et al., 2012	Ethiopia	SNNP, Amahara, Oromia, and Afar Beheira, Gharbia, Giza, and Sharkia governate	2008	Cross-sectional	Endemic	2368	284	0.12	IFAT and VNT
Hodhod et al., 2020	Egypt		2017	Cross-sectional	Endemic	293	260	0.887	RT-PCR
Ochwo et al., 2020	Uganda	Hoima, Moroto, Kotido, and Hoima	2018	Cross-sectional	Outbreak	23	15	0.652	PCR
Dawoud et al., 2019	Egypt	EL-Dakahlia, El-Qalyubia, El-Beheira, and El-Gharbia	2016	Cross-sectional	Endemic	885	210	0.24	PCR and I ELISA
Amin et al., 2021	Egypt	Ismailia, Kafr El-Sheikh, Damitta, and Sharkia	2019	Cross-sectional	Endemic	73	58	0.794	IFAT and PCR
Selim et al., 2021	Egypt	Alexandria, Beheira, Kafr El-Sheikh, Gharbia, and Menofia	2020	Cross-sectional	Outbreak	100	70	0.7	PCR
Mafirakureva et al., 2017	Zimbabwe	Bulawayo, Bindura, Rusape, and Nyanga of Harare	2014	Cross-sectional	Outbreak	25	22	0.88	PCR
Leliso et al., 2021	Ethiopia	Sawena district of Bale zone, Oromiya	2017	Cross-sectional	Outbreak	20	20	1	RT-PCR and c PCR
Mohamed et al., 2022	Egypt	Sharkia governorate	2020	Prospective	Outbreak	61	46	0.75	PCR
Ahmed et al., 2021	Egypt	Assiut, Qaliubiya, Kafer El-Sheikh, and Beheira	2016	Prospective	Outbreak	44	31	0.7045	RT-PCR and c PCR
Overall						10090	1874	0.54	

RT-PCR means real-time PCR; c PCR means conventional PCR.

**Table 2:** Subgroup meta-analysis was done in different variables like country, study design, disease occurrence, and type of diagnostic method.

Group	No. of observations	Prevalence	(95% conf. interval)		*p* value
Country					
Egypt	8	0.584	−0.109	1.277	0.098
Ethiopia	5	0.277	−1.476	2.03	0.757
Uganda	2	0.367	−3.791	4.524	0.863
Zimbabwe	1	0.88	−6.96	8.72	0.826
Study design					
Cross-sectional	12	0.536	−0.238	1.309	0.175
Prospective	3	0.57	−0.562	1.701	0.324
Retrospective	1	0.081	−5.799	5.961	0.978
Disease occurrence					
Endemic	9	0.436	−0.364	1.236	0.285
Outbreak	7	0.719	−0.324	1.761	0.177
Type of diagnostic method					
SNT	1	0.076	−3.844	3.996	0.97
I ELISA	3	0.288	−1.061	1.637	0.675
IFAT	1	0.064	−3.856	3.984	0.974
IFAT and PCR	1	0.794	−1.166	2.754	0.427
IFAT and VNT	2	0.123	−2.649	2.895	0.931
PCR	4	0.726	−0.604	2.055	0.285
PCR and I ELISA	1	0.24	−1.72	2.2	0.81
RT-PCR and c PCR	2	0.764	−0.989	2.517	0.393
RT-PCR	1	0.887	−1.073	2.847	0.375
Overall theta	16	0.541	−0.094	1.176	0.095

**Table 3:** Multivariable meta-regression analysis was calculated in different variables.

Variables	Coefficient	Std. err	*Z*	*P* > *z*	(95% conf. interval)	
Study design	−0.06368	0.788062	−0.08	0.936	−1.608256	1.480888
Disease occurrence	0.257219	0.985135	0.26	0.794	−1.67361	2.188048
Total sample	−0.0005	0.00108	−0.46	0.644	−0.0026142	0.0016176
Event	0.001569	0.006505	0.24	0.809	−0.0111811	0.0143197
Country	0.058889	0.868784	0.07	0.946	−1.643896	1.761675

## Data Availability

The dataset used in this study is available from the corresponding author upon reasonable request.

## Abbreviations

CaPV: Capripoxvirus
DNA: Deoxyribose nucleic acid
ELISA: Enzyme linked immunosorbent assay
Goat Pox Viruses: Goat pox viruses
IFAT: Indirect fluorescent antibody test
GPCR: Gprotein-coupled chemokine receptor
LSDV: Lumpy skin disease viruses
PRISMA: Preferred Reporting Items for Systematic Reviews and Meta-analyses
PCR: Polymerase chain reaction
SPPV: Sheep pox viruses
SNT: Serum neutralization test
VNT: Virus neutralization test.
